# 14a-Hy­droxy-12-methyl-10-(4-methyl­phen­yl)-8,9,9a,10,12,13,14,14a-octa­hydro-10a,14-methano-5*H*-indeno­[2′,1′:4,5]azepino[3,4-*b*]pyrrolizine-5,15(7*H*,11*H*)-dione

**DOI:** 10.1107/S1600536813025592

**Published:** 2013-09-21

**Authors:** R. A. Nagalakshmi, J. Suresh, K. Malathi, R. Ranjith Kumar, P. L. Nilantha Lakshman

**Affiliations:** a Department of Physics, The Madura College, Madurai 625 011, India; b Department of Organic Chemistry, School of Chemistry, Madurai Kamaraj University, Madurai 625 021, India; c Department of Food Science and Technology, University of Ruhuna, Mapalana, Kamburupitiya 81100, Sri Lanka

## Abstract

In the title compound, C_27_H_28_N_2_O_3_, each of the pyrrolidine rings adopts a twisted conformation, as does the cyclo­pentane ring. The indane ring has an r.m.s deviation of 0.0693 Å. The dihedral angle between the mean plane of the pyrrolizine ring and indane system is 82.58 (1)°. The piperidine ring has the methyl substituent in an equatorial position and adopts a twisted chair conformation. The mol­ecular structure is stabilized by a weak intra­molecular O—H⋯N inter­action.

## Related literature

For the importance of pyrrazole derivatives, see: Mahajan *et al.* (1991 ▶); Katayama & Oshiyama (1997 ▶); Baraldi *et al.* (1998 ▶). For additional conformation analysis, see: Cremer & Pople (1975 ▶).
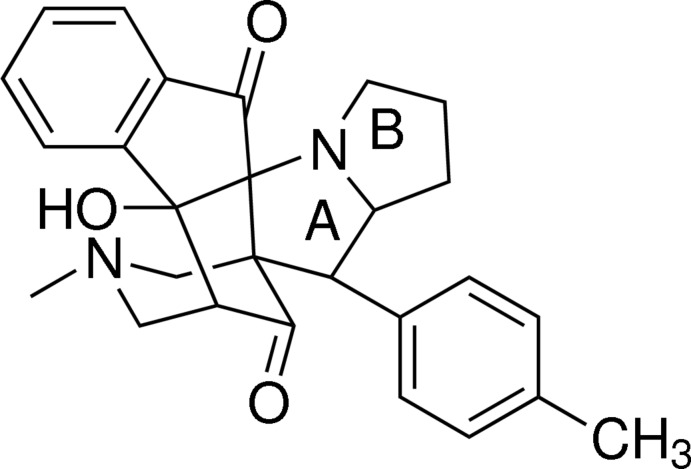


## Experimental

### 

#### Crystal data

C_27_H_28_N_2_O_3_
*M* *_r_* = 428.51
Monoclinic, 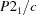

*a* = 16.8684 (6) Å
*b* = 8.3754 (3) Å
*c* = 15.9930 (6) Å
β = 96.658 (1)°
*V* = 2244.25 (14) Å^3^
*Z* = 4
Mo *K*α radiation
μ = 0.08 mm^−1^
*T* = 293 K
0.21 × 0.19 × 0.18 mm

#### Data collection

Bruker Kappa APEXII diffractometer
Absorption correction: multi-scan (*SADABS*; Sheldrick, 1996 ▶) *T*_min_ = 0.967, *T*_max_ = 0.974
28459 measured reflections
6850 independent reflections
4500 reflections with *I* > 2σ(*I*)
*R*_int_ = 0.030

#### Refinement

*R*[*F* ^2^ > 2σ(*F* ^2^)] = 0.053
*wR*(*F* ^2^) = 0.161
*S* = 1.02
6850 reflections
291 parameters
H-atom parameters constrained
Δρ_max_ = 0.29 e Å^−3^
Δρ_min_ = −0.18 e Å^−3^

### 

Data collection: *APEX2* (Bruker, 2004 ▶); cell refinement: *SAINT* (Bruker, 2004 ▶); data reduction: *SAINT*; program(s) used to solve structure: *SHELXS97* (Sheldrick, 2008 ▶); program(s) used to refine structure: *SHELXL97* (Sheldrick, 2008 ▶); molecular graphics: *PLATON* (Spek, 2009 ▶); software used to prepare material for publication: *SHELXL97*.

## Supplementary Material

Crystal structure: contains datablock(s) global, I. DOI: 10.1107/S1600536813025592/tk5254sup1.cif

Structure factors: contains datablock(s) I. DOI: 10.1107/S1600536813025592/tk5254Isup2.hkl

Additional supplementary materials:  crystallographic information; 3D view; checkCIF report

## Figures and Tables

**Table 1:** Hydrogen-bond geometry (Å, °)

*D*—H⋯*A*	*D*—H	H⋯*A*	*D*⋯*A*	*D*—H⋯*A*
O1—H1⋯N2	0.82	2.13	2.6497 (16)	121

Symmetry code: (i) 
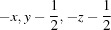
.

**Fig. 1.**
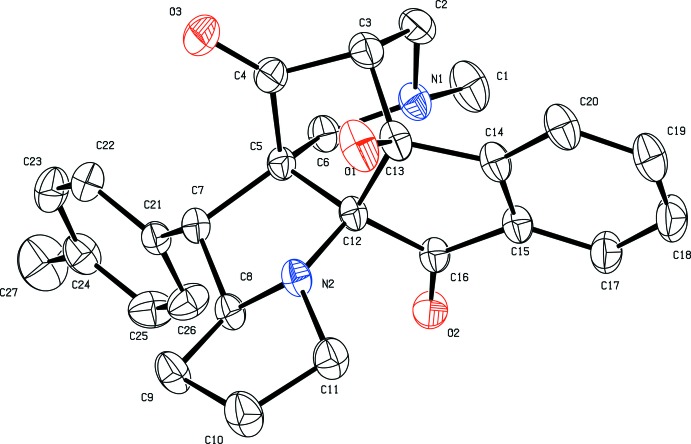
The molecular structure of (I), showing 20% probability displacement ellipsoids and the atom-numbering scheme. H-atoms are omitted for clarity.

## supplementary crystallographic information

### 1. Comment

Pyrazole derivatives in general are well known nitrogen-containing heterocyclic compounds that have been the subject of enormous research due to their importance in various applications and their widespread potential biological and pharmacological activities such as anti-microbial (Mahajan *et al.*, 1991), anti-viral (Baraldi *et al.*, 1998), anti-tumor (Katayama & Oshiyama, 1997) and anti-fungal (Baraldi *et al.*, 1998). In view of its medicinal importance we report the crystal structure of the title compound.

In the title compound (Fig. 1), each of the pyrrolidine rings A and B adopts twisted conformation with the puckering parameters (Cremer & Pople, 1975): q_2_ = 0.4201 (14) Å, Φ_2_ = 10.43 (19)° and q_2_ = 0.4547 (16) Å, Φ_2_ = 196.1 (2)° respectively. The sum of bond angles around N1 (335.69 (2)°) and N2 (340.55 (2)°) indicates *sp*^3^ hybridization. The N2—C11 = 1.462 (12) Å and N2—C8 = 1.463 (17) Å bonds are approximately equivalent and both are longer than the N2—C12 = 1.453 (16) Å bond. The indane (C12—C20) fused-ring system is nearly planar with a r.m.s deviation 0.0693 Å. The plane through the two fused rings of the indane system is slightly folded around the C14—C15 bond, as indicated by the dihedral angle between them of 5.20 (1)°. The dihedral angle between the mean plane of the pyrrolizine and indane system is 82.58 (1)°, indicating that they are nearly perpendicular to each other. The cyclopentane ring adopts a twisted conformation with puckering parameters: q_2_ = 0.4839 (16) Å and Φ_2_ = 17.98 (17)°. The dihedral angle between the mean planes of the pyrrolizine ring system and cyclopentane ring is 58.90 (1)°, indicating the significant curvature between the pyrrolizine and cyclopentene ring systems. The hydroxyl oxygen (O1) attached to the cyclopentane ring deviates by 1.1990 (10) Å from the mean plane of the ring. The piperidine ring with the methyl substituent in an equatorial position, adopts a twisted chair conformation with atoms C2 and C5 deviating by -0.6623 (10) Å and 0.8578 (10) Å respectively, from the mean plane defined by other atoms. In the structure, the aryl ring is in equatorial position of the attached pyrrolidine ring, as indicated by the torsion angle C7—C21—C26—C27 = -178.5 (15)°. The structure features a weak intramolecular O—H···N interaction.

### 2. Experimental

A mixture of 1-methyl-3-[*E*-(4-methylphenyl)methylidene]tetrahydro-2(1*H*)-pyridinone (1 mmol), ninhydrin (1 mmol) and proline (1 mmol) in methanol was refluxed for 3–4 h. After completion of the reaction, as indicated by TLC, the reaction mixture was poured into cold water. The solid precipitate obtained was filtered and dried. The product was purified by column chromatography using petroleum ether:ethylacetate mixture (90:10 *v*/v). Suitable crystals for the single-crystal-X-ray studies were obtained by recrystallizing the product from methanol. Yield: 63%, Melting point: 464–466 K.

### 3. Refinement

H atoms were placed at calculated positions and allowed to ride on their carrier atoms with O—H = 0.82 Å and C—H = 0.93–0.98 Å, and with *U*_iso_ = 1.2*U*_eq_(C) for CH_2_ and CH groups, and *U*_iso_ = 1.5*U*_eq_(O, C) for OH and CH_3_ groups.

### Crystal data

**Table:**

C_27_H_28_N_2_O_3_	*F*(000) = 912
*M**_r_* = 428.51	*D*_x_ = 1.268 Mg m^−^^3^
Monoclinic, *P*2_1_/*c*	Mo *K*α radiation, λ = 0.71073 Å
Hall symbol: -P 2ybc	Cell parameters from 2000 reflections
*a* = 16.8684 (6) Å	θ = 2–31°
*b* = 8.3754 (3) Å	µ = 0.08 mm^−^^1^
*c* = 15.9930 (6) Å	*T* = 293 K
β = 96.658 (1)°	Block, colourless
*V* = 2244.25 (14) Å^3^	0.21 × 0.19 × 0.18 mm
*Z* = 4	

### Data collection

**Table:**

Bruker Kappa APEXII diffractometer	6850 independent reflections
Radiation source: fine-focus sealed tube	4500 reflections with *I* > 2σ(*I*)
Graphite monochromator	*R*_int_ = 0.030
Detector resolution: 0 pixels mm^-1^	θ_max_ = 30.6°, θ_min_ = 2.4°
ω and φ scans	*h* = −24→13
Absorption correction: multi-scan (*SADABS*; Sheldrick, 1996)	*k* = −11→11
*T*_min_ = 0.967, *T*_max_ = 0.974	*l* = −22→22
28459 measured reflections	

### Refinement

**Table:**

Refinement on *F*^2^	Primary atom site location: structure-invariant direct methods
Least-squares matrix: full	Secondary atom site location: difference Fourier map
*R*[*F*^2^ > 2σ(*F*^2^)] = 0.053	Hydrogen site location: inferred from neighbouring sites
*wR*(*F*^2^) = 0.161	H-atom parameters constrained
*S* = 1.02	*w* = 1/[σ^2^(*F*_o_^2^) + (0.0784*P*)^2^ + 0.3451*P*] where *P* = (*F*_o_^2^ + 2*F*_c_^2^)/3
6850 reflections	(Δ/σ)_max_ < 0.001
291 parameters	Δρ_max_ = 0.29 e Å^−^^3^
0 restraints	Δρ_min_ = −0.18 e Å^−^^3^

### Special details

**Table:**

Geometry. All e.s.d.'s (except the e.s.d. in the dihedral angle between two l.s. planes) are estimated using the full covariance matrix. The cell e.s.d.'s are taken into account individually in the estimation of e.s.d.'s in distances, angles and torsion angles; correlations between e.s.d.'s in cell parameters are only used when they are defined by crystal symmetry. An approximate (isotropic) treatment of cell e.s.d.'s is used for estimating e.s.d.'s involving l.s.planes.
Refinement. Refinement of *F*^2^ against ALL reflections. The weighted *R*- factor *wR* and goodness of fit *S* are based on *F*^2^, conventional *R*-factors *R* are based on *F*, with *F* set to zero for negative *F*^2^. The threshold expression of *F*^2^ > σ(*F*^2^) is used only for calculating *R*-factors (gt) *etc*. and is not relevant to the choice of reflections for refinement. *R*-factors based on *F*^2^ are statistically about twice as large as those based on *F*, and *R*-factors based on ALL data will be even larger.

### Fractional atomic coordinates and isotropic or equivalent isotropic displacement parameters (Å^2^)

**Table:**

	*x*	*y*	*z*	*U*_iso_*/*U*_eq_	
C1	0.16397 (13)	−0.0312 (2)	0.12713 (14)	0.0736 (6)	
H1A	0.1240	−0.0997	0.0986	0.110*	
H1B	0.1529	−0.0146	0.1840	0.110*	
H1C	0.2155	−0.0800	0.1274	0.110*	
C2	0.08361 (9)	0.1896 (2)	0.06440 (12)	0.0555 (4)	
H2A	0.0618	0.2147	0.1164	0.067*	
H2B	0.0488	0.1119	0.0338	0.067*	
C3	0.08668 (8)	0.34102 (18)	0.01156 (11)	0.0523 (4)	
H3	0.0337	0.3886	−0.0022	0.063*	
C4	0.14397 (9)	0.45411 (17)	0.06160 (10)	0.0476 (3)	
C5	0.22549 (7)	0.37463 (15)	0.06215 (8)	0.0365 (3)	
C6	0.22196 (9)	0.23342 (17)	0.12322 (9)	0.0436 (3)	
H6A	0.2738	0.1825	0.1337	0.052*	
H6B	0.2064	0.2702	0.1765	0.052*	
C7	0.29914 (8)	0.48839 (15)	0.07586 (8)	0.0381 (3)	
H7	0.2795	0.5984	0.0765	0.046*	
C8	0.33710 (8)	0.46387 (17)	−0.00551 (9)	0.0409 (3)	
H8	0.3715	0.3694	0.0007	0.049*	
C9	0.37893 (11)	0.5938 (2)	−0.04996 (10)	0.0579 (4)	
H9A	0.4353	0.5990	−0.0294	0.069*	
H9B	0.3548	0.6974	−0.0429	0.069*	
C10	0.36594 (13)	0.5389 (2)	−0.14257 (11)	0.0689 (5)	
H10A	0.3475	0.6271	−0.1790	0.083*	
H10B	0.4153	0.4983	−0.1600	0.083*	
C11	0.30260 (10)	0.4065 (2)	−0.14693 (10)	0.0544 (4)	
H11A	0.2626	0.4204	−0.1951	0.065*	
H11B	0.3265	0.3016	−0.1495	0.065*	
C12	0.22046 (7)	0.31215 (15)	−0.03006 (8)	0.0360 (3)	
C13	0.13045 (8)	0.31994 (16)	−0.06716 (10)	0.0452 (3)	
C14	0.11636 (8)	0.16665 (17)	−0.11540 (9)	0.0448 (3)	
C15	0.17990 (8)	0.06209 (17)	−0.09945 (9)	0.0438 (3)	
C16	0.24466 (8)	0.13717 (16)	−0.04209 (9)	0.0396 (3)	
C17	0.17844 (10)	−0.0870 (2)	−0.13690 (12)	0.0576 (4)	
H17	0.2205	−0.1583	−0.1247	0.069*	
C18	0.11302 (11)	−0.1270 (2)	−0.19287 (13)	0.0681 (5)	
H18	0.1113	−0.2261	−0.2193	0.082*	
C19	0.05035 (11)	−0.0227 (2)	−0.21015 (12)	0.0659 (5)	
H19	0.0073	−0.0514	−0.2489	0.079*	
C20	0.05024 (10)	0.1241 (2)	−0.17081 (11)	0.0572 (4)	
H20	0.0069	0.1928	−0.1812	0.069*	
C21	0.35334 (8)	0.46023 (16)	0.15661 (8)	0.0386 (3)	
C22	0.34229 (10)	0.54611 (18)	0.22797 (9)	0.0492 (4)	
H22	0.3025	0.6233	0.2253	0.059*	
C23	0.38944 (12)	0.5195 (2)	0.30366 (10)	0.0588 (4)	
H23	0.3801	0.5786	0.3508	0.071*	
C24	0.44955 (10)	0.4081 (2)	0.31081 (10)	0.0544 (4)	
C25	0.46025 (10)	0.3211 (2)	0.24003 (11)	0.0595 (4)	
H25	0.5002	0.2440	0.2430	0.071*	
C26	0.41302 (10)	0.3457 (2)	0.16452 (10)	0.0554 (4)	
H26	0.4215	0.2841	0.1180	0.066*	
C27	0.50107 (14)	0.3797 (3)	0.39303 (13)	0.0907 (7)	
H27A	0.5197	0.2713	0.3952	0.136*	
H27B	0.4704	0.3987	0.4390	0.136*	
H27C	0.5459	0.4510	0.3973	0.136*	
N1	0.16329 (7)	0.12170 (14)	0.08371 (8)	0.0466 (3)	
N2	0.26863 (7)	0.42998 (14)	−0.06780 (7)	0.0412 (3)	
O1	0.11417 (7)	0.45503 (13)	−0.11974 (9)	0.0661 (4)	
H1	0.1531	0.5144	−0.1152	0.099*	
O2	0.30850 (6)	0.07901 (13)	−0.01635 (7)	0.0495 (3)	
O3	0.12969 (7)	0.57851 (14)	0.09413 (9)	0.0683 (4)	

### Atomic displacement parameters (Å^2^)

**Table:**

	*U*^11^	*U*^22^	*U*^33^	*U*^12^	*U*^13^	*U*^23^
C1	0.0835 (13)	0.0447 (9)	0.0899 (14)	−0.0161 (9)	−0.0015 (11)	0.0194 (9)
C2	0.0445 (8)	0.0507 (9)	0.0722 (11)	−0.0076 (7)	0.0108 (7)	0.0000 (8)
C3	0.0346 (6)	0.0406 (8)	0.0802 (11)	0.0045 (6)	0.0001 (7)	−0.0030 (7)
C4	0.0462 (7)	0.0355 (7)	0.0611 (9)	0.0035 (6)	0.0065 (7)	0.0001 (6)
C5	0.0383 (6)	0.0290 (6)	0.0413 (7)	−0.0008 (5)	0.0010 (5)	−0.0004 (5)
C6	0.0487 (7)	0.0373 (7)	0.0440 (7)	−0.0039 (6)	0.0026 (6)	0.0042 (6)
C7	0.0417 (6)	0.0314 (6)	0.0398 (7)	−0.0031 (5)	−0.0011 (5)	−0.0006 (5)
C8	0.0429 (7)	0.0407 (7)	0.0374 (7)	−0.0081 (5)	−0.0024 (5)	0.0013 (5)
C9	0.0632 (10)	0.0599 (10)	0.0499 (9)	−0.0257 (8)	0.0036 (7)	0.0054 (7)
C10	0.0879 (13)	0.0713 (12)	0.0489 (10)	−0.0298 (10)	0.0138 (9)	0.0033 (8)
C11	0.0668 (10)	0.0559 (9)	0.0396 (8)	−0.0119 (8)	0.0028 (7)	0.0005 (7)
C12	0.0364 (6)	0.0301 (6)	0.0396 (7)	−0.0022 (5)	−0.0043 (5)	0.0025 (5)
C13	0.0396 (7)	0.0326 (6)	0.0592 (9)	0.0005 (5)	−0.0119 (6)	0.0076 (6)
C14	0.0448 (7)	0.0394 (7)	0.0471 (8)	−0.0074 (6)	−0.0087 (6)	0.0053 (6)
C15	0.0438 (7)	0.0394 (7)	0.0462 (8)	−0.0046 (6)	−0.0037 (6)	−0.0021 (6)
C16	0.0408 (7)	0.0359 (7)	0.0405 (7)	−0.0012 (5)	−0.0019 (5)	−0.0007 (5)
C17	0.0548 (9)	0.0479 (9)	0.0674 (11)	−0.0029 (7)	−0.0046 (8)	−0.0152 (8)
C18	0.0704 (11)	0.0599 (11)	0.0704 (12)	−0.0127 (9)	−0.0077 (9)	−0.0229 (9)
C19	0.0642 (10)	0.0682 (12)	0.0593 (10)	−0.0210 (9)	−0.0177 (8)	−0.0066 (9)
C20	0.0521 (8)	0.0527 (9)	0.0608 (10)	−0.0091 (7)	−0.0187 (7)	0.0091 (8)
C21	0.0394 (6)	0.0376 (7)	0.0382 (7)	−0.0056 (5)	0.0023 (5)	−0.0020 (5)
C22	0.0598 (9)	0.0427 (8)	0.0446 (8)	0.0016 (7)	0.0035 (7)	−0.0055 (6)
C23	0.0833 (12)	0.0543 (9)	0.0377 (8)	−0.0053 (9)	0.0018 (8)	−0.0079 (7)
C24	0.0524 (8)	0.0644 (10)	0.0436 (8)	−0.0118 (8)	−0.0058 (7)	0.0055 (7)
C25	0.0476 (8)	0.0773 (12)	0.0526 (9)	0.0129 (8)	0.0014 (7)	0.0074 (8)
C26	0.0552 (9)	0.0691 (11)	0.0412 (8)	0.0156 (8)	0.0027 (7)	−0.0045 (7)
C27	0.0929 (15)	0.1150 (19)	0.0560 (12)	−0.0076 (14)	−0.0256 (11)	0.0093 (12)
N1	0.0495 (7)	0.0351 (6)	0.0547 (7)	−0.0066 (5)	0.0038 (6)	0.0059 (5)
N2	0.0464 (6)	0.0390 (6)	0.0364 (6)	−0.0089 (5)	−0.0027 (5)	0.0026 (5)
O1	0.0616 (7)	0.0435 (6)	0.0851 (9)	−0.0024 (5)	−0.0266 (6)	0.0229 (6)
O2	0.0404 (5)	0.0462 (6)	0.0591 (6)	0.0073 (4)	−0.0057 (5)	−0.0043 (5)
O3	0.0640 (7)	0.0453 (6)	0.0966 (10)	0.0087 (5)	0.0137 (7)	−0.0168 (6)

### Geometric parameters (Å, º)

**Table:**

C1—N1	1.456 (2)	C11—H11B	0.9700
C1—H1A	0.9600	C12—N2	1.4533 (17)
C1—H1B	0.9600	C12—C16	1.5394 (18)
C1—H1C	0.9600	C12—C13	1.5661 (18)
C2—N1	1.459 (2)	C13—O1	1.4174 (17)
C2—C3	1.528 (2)	C13—C14	1.503 (2)
C2—H2A	0.9700	C14—C15	1.385 (2)
C2—H2B	0.9700	C14—C20	1.3881 (19)
C3—C4	1.514 (2)	C15—C17	1.384 (2)
C3—C13	1.542 (2)	C15—C16	1.4825 (19)
C3—H3	0.9800	C16—O2	1.2098 (16)
C4—O3	1.2014 (18)	C17—C18	1.379 (2)
C4—C5	1.5269 (19)	C17—H17	0.9300
C5—C6	1.5394 (18)	C18—C19	1.375 (3)
C5—C12	1.5578 (18)	C18—H18	0.9300
C5—C7	1.5608 (17)	C19—C20	1.382 (3)
C6—N1	1.4524 (18)	C19—H19	0.9300
C6—H6A	0.9700	C20—H20	0.9300
C6—H6B	0.9700	C21—C22	1.380 (2)
C7—C21	1.5118 (18)	C21—C26	1.385 (2)
C7—C8	1.5293 (19)	C22—C23	1.387 (2)
C7—H7	0.9800	C22—H22	0.9300
C8—N2	1.4629 (17)	C23—C24	1.373 (3)
C8—C9	1.518 (2)	C23—H23	0.9300
C8—H8	0.9800	C24—C25	1.375 (2)
C9—C10	1.542 (2)	C24—C27	1.509 (2)
C9—H9A	0.9700	C25—C26	1.383 (2)
C9—H9B	0.9700	C25—H25	0.9300
C10—C11	1.536 (2)	C26—H26	0.9300
C10—H10A	0.9700	C27—H27A	0.9600
C10—H10B	0.9700	C27—H27B	0.9600
C11—N2	1.462 (2)	C27—H27C	0.9600
C11—H11A	0.9700	O1—H1	0.8200
			
N1—C1—H1A	109.5	N2—C12—C5	101.15 (10)
N1—C1—H1B	109.5	C16—C12—C5	116.87 (11)
H1A—C1—H1B	109.5	N2—C12—C13	112.24 (10)
N1—C1—H1C	109.5	C16—C12—C13	104.58 (10)
H1A—C1—H1C	109.5	C5—C12—C13	106.78 (11)
H1B—C1—H1C	109.5	O1—C13—C14	111.67 (12)
N1—C2—C3	110.55 (12)	O1—C13—C3	108.35 (12)
N1—C2—H2A	109.5	C14—C13—C3	117.05 (12)
C3—C2—H2A	109.5	O1—C13—C12	111.76 (11)
N1—C2—H2B	109.5	C14—C13—C12	104.34 (11)
C3—C2—H2B	109.5	C3—C13—C12	103.32 (11)
H2A—C2—H2B	108.1	C15—C14—C20	120.18 (14)
C4—C3—C2	106.70 (13)	C15—C14—C13	111.89 (11)
C4—C3—C13	99.58 (12)	C20—C14—C13	127.93 (14)
C2—C3—C13	113.99 (12)	C17—C15—C14	121.09 (13)
C4—C3—H3	111.9	C17—C15—C16	128.71 (14)
C2—C3—H3	111.9	C14—C15—C16	110.17 (12)
C13—C3—H3	111.9	O2—C16—C15	127.06 (13)
O3—C4—C3	128.58 (14)	O2—C16—C12	125.40 (12)
O3—C4—C5	126.97 (14)	C15—C16—C12	107.10 (11)
C3—C4—C5	104.45 (11)	C18—C17—C15	118.22 (16)
C4—C5—C6	103.74 (11)	C18—C17—H17	120.9
C4—C5—C12	101.00 (11)	C15—C17—H17	120.9
C6—C5—C12	109.92 (10)	C19—C18—C17	120.98 (17)
C4—C5—C7	115.87 (11)	C19—C18—H18	119.5
C6—C5—C7	117.94 (11)	C17—C18—H18	119.5
C12—C5—C7	107.05 (10)	C18—C19—C20	121.05 (15)
N1—C6—C5	107.06 (11)	C18—C19—H19	119.5
N1—C6—H6A	110.3	C20—C19—H19	119.5
C5—C6—H6A	110.3	C19—C20—C14	118.41 (16)
N1—C6—H6B	110.3	C19—C20—H20	120.8
C5—C6—H6B	110.3	C14—C20—H20	120.8
H6A—C6—H6B	108.6	C22—C21—C26	116.85 (13)
C21—C7—C8	115.76 (11)	C22—C21—C7	119.93 (13)
C21—C7—C5	114.67 (11)	C26—C21—C7	123.17 (13)
C8—C7—C5	101.74 (10)	C21—C22—C23	121.27 (15)
C21—C7—H7	108.1	C21—C22—H22	119.4
C8—C7—H7	108.1	C23—C22—H22	119.4
C5—C7—H7	108.1	C24—C23—C22	121.68 (15)
N2—C8—C9	101.15 (11)	C24—C23—H23	119.2
N2—C8—C7	103.31 (11)	C22—C23—H23	119.2
C9—C8—C7	124.43 (13)	C23—C24—C25	117.22 (15)
N2—C8—H8	108.9	C23—C24—C27	121.66 (18)
C9—C8—H8	108.9	C25—C24—C27	121.11 (18)
C7—C8—H8	108.9	C24—C25—C26	121.50 (16)
C8—C9—C10	102.34 (12)	C24—C25—H25	119.3
C8—C9—H9A	111.3	C26—C25—H25	119.3
C10—C9—H9A	111.3	C25—C26—C21	121.46 (15)
C8—C9—H9B	111.3	C25—C26—H26	119.3
C10—C9—H9B	111.3	C21—C26—H26	119.3
H9A—C9—H9B	109.2	C24—C27—H27A	109.5
C11—C10—C9	106.25 (13)	C24—C27—H27B	109.5
C11—C10—H10A	110.5	H27A—C27—H27B	109.5
C9—C10—H10A	110.5	C24—C27—H27C	109.5
C11—C10—H10B	110.5	H27A—C27—H27C	109.5
C9—C10—H10B	110.5	H27B—C27—H27C	109.5
H10A—C10—H10B	108.7	C6—N1—C1	113.06 (13)
N2—C11—C10	101.77 (12)	C6—N1—C2	113.93 (12)
N2—C11—H11A	111.4	C1—N1—C2	113.56 (13)
C10—C11—H11A	111.4	C12—N2—C11	124.22 (11)
N2—C11—H11B	111.4	C12—N2—C8	106.51 (10)
C10—C11—H11B	111.4	C11—N2—C8	104.95 (11)
H11A—C11—H11B	109.3	C13—O1—H1	109.5
N2—C12—C16	115.18 (11)		
			
N1—C2—C3—C4	−57.27 (17)	C12—C13—C14—C15	10.41 (16)
N1—C2—C3—C13	51.62 (18)	O1—C13—C14—C20	−48.4 (2)
C2—C3—C4—O3	−111.93 (19)	C3—C13—C14—C20	77.3 (2)
C13—C3—C4—O3	129.31 (18)	C12—C13—C14—C20	−169.26 (15)
C2—C3—C4—C5	67.92 (15)	C20—C14—C15—C17	−1.1 (2)
C13—C3—C4—C5	−50.84 (14)	C13—C14—C15—C17	179.19 (15)
O3—C4—C5—C6	106.79 (18)	C20—C14—C15—C16	177.07 (14)
C3—C4—C5—C6	−73.07 (14)	C13—C14—C15—C16	−2.63 (18)
O3—C4—C5—C12	−139.33 (17)	C17—C15—C16—O2	−1.3 (3)
C3—C4—C5—C12	40.81 (14)	C14—C15—C16—O2	−179.28 (14)
O3—C4—C5—C7	−24.1 (2)	C17—C15—C16—C12	171.45 (16)
C3—C4—C5—C7	156.03 (12)	C14—C15—C16—C12	−6.55 (16)
C4—C5—C6—N1	67.25 (14)	N2—C12—C16—O2	61.65 (19)
C12—C5—C6—N1	−40.06 (14)	C5—C12—C16—O2	−56.89 (19)
C7—C5—C6—N1	−163.10 (11)	C13—C12—C16—O2	−174.68 (14)
C4—C5—C7—C21	115.03 (14)	N2—C12—C16—C15	−111.23 (12)
C6—C5—C7—C21	−8.76 (17)	C5—C12—C16—C15	130.23 (12)
C12—C5—C7—C21	−133.23 (11)	C13—C12—C16—C15	12.44 (14)
C4—C5—C7—C8	−119.25 (13)	C14—C15—C17—C18	2.1 (3)
C6—C5—C7—C8	116.97 (13)	C16—C15—C17—C18	−175.70 (16)
C12—C5—C7—C8	−7.51 (13)	C15—C17—C18—C19	−0.9 (3)
C21—C7—C8—N2	156.36 (11)	C17—C18—C19—C20	−1.3 (3)
C5—C7—C8—N2	31.36 (12)	C18—C19—C20—C14	2.3 (3)
C21—C7—C8—C9	−89.87 (17)	C15—C14—C20—C19	−1.1 (2)
C5—C7—C8—C9	145.12 (13)	C13—C14—C20—C19	178.57 (16)
N2—C8—C9—C10	−36.92 (17)	C8—C7—C21—C22	148.72 (13)
C7—C8—C9—C10	−151.73 (15)	C5—C7—C21—C22	−93.23 (15)
C8—C9—C10—C11	12.7 (2)	C8—C7—C21—C26	−34.09 (19)
C9—C10—C11—N2	16.2 (2)	C5—C7—C21—C26	83.96 (17)
C4—C5—C12—N2	102.67 (11)	C26—C21—C22—C23	0.6 (2)
C6—C5—C12—N2	−148.20 (11)	C7—C21—C22—C23	177.97 (14)
C7—C5—C12—N2	−18.97 (12)	C21—C22—C23—C24	0.7 (3)
C4—C5—C12—C16	−131.46 (12)	C22—C23—C24—C25	−1.3 (3)
C6—C5—C12—C16	−22.32 (15)	C22—C23—C24—C27	179.71 (18)
C7—C5—C12—C16	106.91 (12)	C23—C24—C25—C26	0.6 (3)
C4—C5—C12—C13	−14.88 (12)	C27—C24—C25—C26	179.64 (19)
C6—C5—C12—C13	94.26 (12)	C24—C25—C26—C21	0.7 (3)
C7—C5—C12—C13	−136.51 (10)	C22—C21—C26—C25	−1.3 (2)
C4—C3—C13—O1	−79.52 (13)	C7—C21—C26—C25	−178.53 (15)
C2—C3—C13—O1	167.27 (12)	C5—C6—N1—C1	169.39 (14)
C4—C3—C13—C14	153.16 (12)	C5—C6—N1—C2	−58.99 (16)
C2—C3—C13—C14	39.95 (17)	C3—C2—N1—C6	54.13 (18)
C4—C3—C13—C12	39.16 (13)	C3—C2—N1—C1	−174.49 (15)
C2—C3—C13—C12	−74.05 (14)	C16—C12—N2—C11	35.17 (18)
N2—C12—C13—O1	−8.82 (17)	C5—C12—N2—C11	162.16 (13)
C16—C12—C13—O1	−134.36 (13)	C13—C12—N2—C11	−84.36 (16)
C5—C12—C13—O1	101.15 (14)	C16—C12—N2—C8	−86.64 (13)
N2—C12—C13—C14	112.01 (12)	C5—C12—N2—C8	40.35 (13)
C16—C12—C13—C14	−13.54 (14)	C13—C12—N2—C8	153.84 (12)
C5—C12—C13—C14	−138.02 (11)	C10—C11—N2—C12	−163.50 (14)
N2—C12—C13—C3	−125.10 (12)	C10—C11—N2—C8	−40.99 (16)
C16—C12—C13—C3	109.35 (12)	C9—C8—N2—C12	−176.64 (12)
C5—C12—C13—C3	−15.13 (13)	C7—C8—N2—C12	−46.94 (13)
O1—C13—C14—C15	131.29 (14)	C9—C8—N2—C11	50.03 (15)
C3—C13—C14—C15	−103.02 (15)	C7—C8—N2—C11	179.73 (11)

### Hydrogen-bond geometry (Å, º)

**Table:**

*D*—H···*A*	*D*—H	H···*A*	*D*···*A*	*D*—H···*A*
O1—H1···N2	0.82	2.13	2.6497 (16)	121
C19—H19···O1^i^	0.93	2.76	3.659 (2)	163

Symmetry code: (i) −*x*, *y*−1/2, −*z*−1/2.

## References

[bb1] Baraldi, P. G., Manfredini, S., Romagnoli, R., Stevanato, L., Zaid, A. N. & Manservigi, R. (1998). *Nucleosides Nucleotides*, **17**, 2165–2171.

[bb2] Bruker (2004). *APEX2* and *SAINT* Bruker AXS Inc., Madison, Wisconsin, USA.

[bb3] Cremer, D. & Pople, J. A. (1975). *J. Am. Chem. Soc.* **97**, 1354–1358.

[bb4] Katayama, H. & Oshiyama, T. (1997). *Can. J. Chem.* **75**, 913–919.

[bb5] Mahajan, R. N., Havaldar, F. H. & Fernandes, P. S. (1991). *J. Indian Chem. Soc.* **68**, 245–249.

[bb6] Sheldrick, G. M. (1996). *SADABS*, University of Göttingen, Germany.

[bb7] Sheldrick, G. M. (2008). *Acta Cryst.* A**64**, 112–122. 10.1107/S0108767307043930 18156677

[bb8] Spek, A. L. (2009). *Acta Cryst.* D**65**, 148–155. 10.1107/S090744490804362X PMC2631630 19171970

